# The association between obesity-related legislation in the United States and adolescents’ weight

**DOI:** 10.1016/j.hpopen.2021.100056

**Published:** 2021-12-01

**Authors:** Annita Kobes, Tina Kretschmer, Margaretha C. Timmerman

**Affiliations:** University of Groningen, Faculty of Behavioural and Social Sciences, Department of Pedagogical and Educational Sciences, Groningen 9712TJ, the Netherlands

**Keywords:** State-level policy, Obesity-related legislation, Obesity prevalence, BMI, Adolescents

## Abstract

Effective obesity prevention requires intervening at all levels of society, including the governmental level. Policy interventions at the governmental level are especially promising as they tend to involve minimal individual effort while, at the same time, reach many people. The amount of state-level obesity-related legislation in the United States has increased over the years, and several laws were installed in contexts that reach young people, such as schools. Given this increase in state-level obesity-related legislation targeting youth, we tested whether the quantity of obesity-related legislation in U.S. states was associated with adolescent BMI and overweight/obesity prevalence. Linear and multilevel analyses showed that the quantity of physical activity-related legislation was associated with lower overweight/obesity prevalence yet with very modest effect size (*b* = -0.002, *p* = .042). Our results underline the likely importance of obesity-related legislation. In addition, the value of examining both BMI and overweight/obesity prevalence when evaluating interventions is demonstrated.

## Highlights

• Overall, state-level obesity-related legislation was not significantly associated with adolescents’ average BMI.
• Exploratory analyses showed that physical activity-related legislation was associated with overweight/obesity prevalence.
• Findings emphasize the importance of obesity-related legislation for reducing obesity prevalence.

## 1 Introduction

Obesity is affecting more than 13 million youth under the age of 19 in the United States [1], and the short-term and long-term risks and consequences of childhood obesity are well-described [2], [3]. Effective obesity-related policy requires intervening at all levels of society, including at the governmental level, and necessitates the involvement of multiple sectors and stakeholders to create a healthier environment. In line, recent efforts to combat childhood obesity have focused not only on the individual but also on their surroundings [4]. An obesogenic environment impedes the choice of healthful behaviors. In the past 30 years, two developments have resulted in the obesogenic environment that many children and adolescents now live in: the increasing availability of high-calorie food and beverages, and the emergence of technology that facilitates sedentary behavior (e.g., internet, video gaming). To modify the obesogenic environment with respect to nutrition and physical activity, intervening at the policy-level is considered inevitable [5], [6], [7].

Policy interventions are especially promising as they require minimal individual effort while at the same time reach many people [8]. Politicians, local governments and other stakeholders at policy-level are urged to implement interventions that facilitate and promote making healthier choices [9], and several policy-level interventions have already resulted in improved health outcomes. Legislation that imposes a sales tax on sugar-sweetened beverages to discourage consumers to buy these drinks, and to encourage producers to create alternatives that contain less sugar [10] is just one example. “Sugar taxes” have been associated with decreased BMI [11] and reduced soda consumption [12] among adolescents. U.S. state laws also regulate “competitive foods” in schools, which are products sold outside of federal meal programs, such as snacks sold through vending machines or school stores. For a long time, competitive foods did not need to comply with regulations concerning the nutritional value and contents of foods and generally contained high levels of sugar and fat [13], [14]. States that installed legislation to regulate the nutritional value of competitive foods showed a decrease in adolescents’ BMI [15], [16]. Likewise, the Safe Routes to School program, adopted by all U.S. states, supports the improvement of sidewalks, bike paths, and safe street crossings and resulted in increased numbers of youth walking or bicycling to school [17]. These examples show that implementing interventions at policy-level can help in creating environments that facilitate choosing health-improving behaviors over health-impairing behaviors, and contribute to modifying the obesogenic environment.

The number of obesity-related laws that were introduced and adopted by states in the U.S. has increased over the years [18], [19], [20], and the largest numbers of laws were introduced in the school environment, community environment, and health care environment [19]. Likewise, between 1992 and 2006, the amount of funding spent on obesity-related legislation rose from $660k for seven states to nearly $248 million for all states [21]. These growing numbers of obesity-related legislation and obesity-related funding show that governments increasingly invest time and money into obesity prevention and treatment from the level of the policy. Given this growing attention for obesity-related legislation, we examined whether the increased number of obesity-related legislation can be associated with better, i.e., more healthful, weight-outcomes.

Previous research on links between the number of active state-level legislation and weight status has found no statistically significant associations [18], [22]. However, these studies examined the association between state-level laws and adult BMI. However, school nutrition standards, physical education and activity regulations, and Safe Routes to School programs [18] – i.e., child-related adjustments - were among the most frequently installed obesity-related laws. Given that the environments of youth are more often the target of obesity-related legislation, we hypothesized that obesity-related legislation would be more effective for youth than was previously found for adults. To this end, we examined associations between obesity-related policy making in U.S. states and adolescents’ BMI z-score by employing anthropometric data obtained in 2017 from the Youth Risk Behavior Survey (YRBS) and publicly available data on obesity-related legislation in U.S. states. In line with previous research [22], we hypothesized that a greater number of active laws can be seen as an expression of *greater* effort to modify the obesogenic environment and are expected to be associated with lower BMI z-score among adolescents. Additionally, we tested whether this association was stronger when taking into account only laws that explicitly target youth, e.g., concern school nutrition policy or after-school physical activity legislation. Finally, it might take some time for a law to have an effect on health outcomes. Adhering to previous investigations that took into account a one-to-three-year-lag period [12], [22], we analyzed the association between obesity-related legislation implemented before 2015 and adolescents’ BMI, thereby accounting for a three-year-lag period.

## 2 Materials and methods

### 2.1 Procedure and participants

The Youth Risk Behavior Surveillance System (YRBS) conducts biannual school-based surveys to gain insight in the health-related behaviors of youth. For the 2017 state-level YRBS, samples of high school students in grades 9 to 12 were drawn and surveyed according to a two-stage cluster design during February-May. The sampling design has been described in detail elsewhere [23]. Students completed questionnaires voluntarily and anonymously during school hours. Data were weighted to adjust for school and student non-response, as well as the oversampling of black and Hispanic students, and are representative at state-level. Surveys of adolescents that did not complete ≥60% of the questionnaires were not weighted, and unweighted data were not made publicly available. Data can be retrieved from the YRBS website [24].

In 2017, the sample consisted of 107,664 adolescents from 33 states. Data of 17 states could not be weighted and were thus not released as publicly available data. Of the 107,664 adolescents, 54,106 (50.25%) were girls and 52,444 (48.71%) were boys; 1,114 adolescents did not report their sex (1.03%). Adolescents were aged 12 to 18 with a mean age of 15.88 years (*SD* = 1.24). The majority of adolescents (51.42%) described themselves as White, 20.1% described themselves as Hispanic or Latino, and 10.37% as Black or African American. 15.08% identified as other ethnicities, and 3.03% did not report their ethnicity.

### 2.2 Measures

***BMI z-score.*** Since 1999, participants were asked how tall they were without their shoes on, measured in feet and inches, and how much they weighed without their shoes on, measured in pounds. Height and weight were converted to centimeters and kilograms by the Centers for Disease Control and Prevention (CDC) and translated to BMI, and we converted these scores to age-specific BMI z-scores. The adolescents’ age-range of 12 to 18 meant that we used a standardized anthropometric score instead of regular BMI, because regular BMI is interpreted differently for different ages. For example, a 13-year-old boy with a BMI of 23.8 would be considered overweight, while a 17-year-old girl with a BMI of 23.8 would be considered healthy weight. According to CDC recommendations, a BMI z-score of 1.04 indicates overweight, while a z-score of 1.64 indicates obesity [25]. These cut-off scores correspond to a BMI of 25 for adults and a BMI of 30 for adults, respectively. For this sample, we used the CDC reference standard. BMIz was used as dependent variable.

***Legislation.*** The CDC published policy data for U.S. states from 2001 to 2017 including information on: a) the state in which a law was implemented and the exact geographical location; b) in which year a law was implemented and the exact enactment date; c) the health topic the law pertains to (i.e., nutrition, obesity, physical activity); d) the policy topic it fits in (e.g., media campaigns, food restrictions, initiatives and programs); e) the setting it was implemented in (e.g., community, school/after school, work place); f) the law’s title and corresponding title code; and g) the law’s status, i.e., introduced (a law that is drafted and introduced to the appropriate legislative body), dead (an introduced law that is not further acted upon by legislators), vetoed (a law that is opposed by the state Governor), or enacted (an active law). For every law, a short description clarifies the law’s content, e.g., “Creates the Healthy Food Financing Act to provide financing for grocery stores to operate in low income areas and food deserts in order to increase the availability of nutritious food to underserved communities. Appropriates monies to the Healthy Food Financing Fund consisting of federal, state, and private loans” [26].

**Fig. 1:**
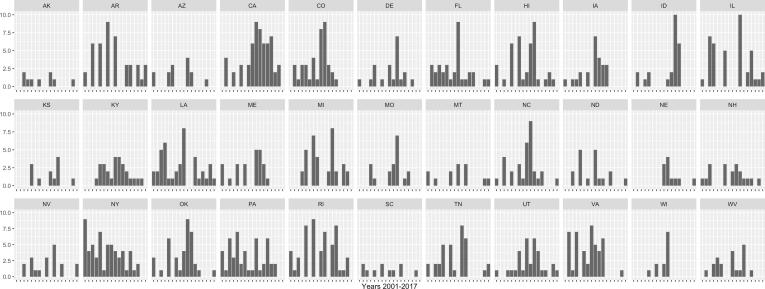
Overview of the number of laws per state. Note. Each bar represents the number of laws that were installed in that specific year (2001-2017).

After removing laws that were not enacted from the data file, the CDC file contained 5,859 laws in 50 states, however, these were not all unique laws. Laws with equal title codes and titles that were implemented in the same state and same year and quarter, and that shared the exact enactment date and geolocation, were considered definite duplicates. A total of 1,475 unique enacted laws remained for the 33 states for which YRBS information was available (Fig. 1).

We categorized laws into four different groups based on their title and description: 1) laws that pertained to nutrition, such as laws that concerned food and drinks at schools or child care facilities, food labeling, or nutritional information on menus (*n* = 524); 2) laws that pertained to physical activity, such as laws that concerned the amount of mandatory physical exercise at schools, or Safe Routes to School initiatives (*n* = 464); 3) laws that pertained to physical activity *and* nutrition, or obesity in general. This latter category includes, for example, laws that govern the establishment of a “fitness month” in which citizens of a state are actively encouraged to enrich their lives through healthy diet and exercise, or laws that fund interventions to decrease obesity (*n* = 188); 4) laws that did not explicitly concern nutrition, physical activity, or obesity in general, such as laws that installed a committee to promote the lease of land for use of beginning farmers (*n* = 299). Furthermore, we categorized laws into two target groups: a) laws that focused explicitly on minors, i.e., targeting schools, child care facilities, or playgrounds (*n* = 436), and b) laws targeting the general audience, such as a law that forced restaurants to mention nutritional information on their menus. These laws could affect adolescents but were not explicitly targeting them (*n* = 1039).

### 2.3 Analytic procedure

For every state, we created four summary variables. Variable *p0* expressed the total number of all obesity-related legislation that was installed between 2001 and 2017; variable *p1* expressed the total number of nutrition-related laws between 2001 and 2017; variable *p2* expressed the total number of physical activity-related laws between 2001 and 2017; and variable *p3* expressed the total number of physical activity- *and* nutrition-related laws, or laws that pertained to obesity in general. This is called the *combined* category from here on; and variable *p4* expressed the total number of laws that do not explicitly concern nutrition, physical activity, or obesity in general.

We used multilevel regression models to analyze the data with BMI z-score as dependent variable and variables *p0* to *p4* as predictor variables. Using a multilevel model allowed us to include BMI z-score as an individual-level variable and to preserve all variance in the dependent variable, while including predictors at the group-level. Four models were estimated: 1) *Model 1* estimated the effect of the total number of laws *p0* on BMI z-score; 2) *Model 2: p1*, *p2*, *p3* and *p4* were included as predictor variables to estimate their effect on BMI z-score; 3) *Model 3:* Similar to model 2, estimated the effects of *p1, p2, p3,* and *p4* on BMIz, however, the predictor variables in this model consisted of only legislation specifically targeting youth; 4) *Model 4:* Similar to model 2, we estimated the effects of *p1, p2, p3,* and *p4* on BMIz. However, the predictor variables in model 4 included only laws that were implemented before 2015.

Theoretically, random intercepts and slopes models should represent the data used here most optimally as the effect of the predictor variables might vary between states. However, estimating random intercept and slope models in “lme4” in R [27] returned a singular fit warning, which implies that the variance of one or more effects is estimated near zero. Therefore, we estimated random intercept and fixed slopes models instead, and compared the results of these models to the results of the model with random intercepts and slopes as sensitivity check. Furthermore, the dependent variable contained missing information of 9.2% of BMI z-scores. Missing information would impact our results if adolescents in states with high prevalence of overweight/obesity were less likely to report their height and weight. To test this, we computed the correlation between the prevalence of overweight/obesity in a state and the proportion of missing data in a state (*r* = 0.48, *p* = .004), showing that the proportion of missing data was smaller in states with high prevalence of overweight/obesity than in states with low prevalence of overweight/obesity. To adjust for missing data bias, we used multiple imputation. Data was imputed twenty times, and analyses were conducted in R using the package “mice” [28]. We compared the results of these analyses to results obtained with multilevel analyses on the original data to understand the impact of the missing values on BMI z-score. Finally, we conducted exploratory analyses with proportion overweight/obesity as dependent variable instead of BMI z-score. We conducted these exploratory analyses, because previous research has shown that patterns over time of average BMI and prevalence of overweight/obesity are not necessarily equivalent [29], [30]. Given that the proportion overweight/obesity is a state-level variable, and so are the predictor variables, we conducted linear regression models instead of multilevel models. We applied a false discovery rate approach to adjust for multiple comparisons [31].

## 3 Results

### 3.1 Descriptive statistics

BMIz was 0.54 on average (*SD* = 1.06), and was within the boundaries for healthy weight, i.e., BMIz −1.64 to 1.04. Overweight/obesity prevalence was 32.60% among all adolescents, and higher among boys than among girls (*p* < .001). Average BMIz was 0.57 for boys and 0.51 for girls. Average BMIz differed significantly between age categories (*p* < .001), and was highest among 12-year-olds and indicated overweight status (*M_BMIz_* = 1.33, *SD* = 0.93), but overweight/obesity prevalence was lowest in this group: 18.74%. A possible explanation for this result is that relatively many 12-year-old children scored very highly on BMIz. BMIz was lowest among 18-year-olds (*M_BMIz_* = 0.46, *SD* = 1.14), and prevalence of overweight/obesity was 28.93%.

### 3.2 Association between obesity-related laws and BMI

None of the predictor variables, that is, the total number of enacted laws (*p0*), nutrition-related legislation (*p1*), physical activity-related legislation (*p2*), combined legislation (*p3*), or other legislation (*p4*) showed a statistically significant effect on BMIz (Table 1). Solely physical activity-related laws showed a trend towards a decreasing effect on BMIz (*p* = .066), suggesting that for every additional physical activity-related law, the average BMIz among children and adolescents would decrease by 0.005. Table 1 also shows that including only laws that were implemented before 2015 did not result in different effects on BMIz. Comparisons with the results of random intercepts and random slopes models presented no different results that would lead to other conclusions (Table S1). Comparisons with multilevel regression analyses conducted without multiple imputation showed that results were similar to the extent that the results of analyses on non-imputed data did not lead to different conclusions (Table S2).

**Table 1:** Results of random intercepts models with imputed data.

	Model 1			Model 2			Model 3			Model 4		
	Est	SE	p-value	Est	SE	p-value	Est	SE	p-value	Est	SE	p-value
Intercept	0.551	0.031	<0.001	0.551	0.032	<0.001	0.547	0.028	<0.001	0.551	0.032	<0.001
P0: Total	0.000	0.001	0.733									
P1: Nutrition				0.004	0.003	0.149	−0.005	0.005	0.284	0.004	0.003	0.150
P2: PA				−0.005	0.003	0.066	0.003	0.007	0.634	−0.005	0.003	0.063
P3: Combined				0.007	0.006	0.267	0.006	0.007	0.408	0.007	0.006	0.269
P4: Other				−0.003	0.004	0.461	0.013	0.033	0.689	−0.003	0.004	0.479

*Note*. Model 1 estimated the effect of the total number of laws p0 on BMI z-score; Model 2 estimated the effects of p1, p2, p3, and p4 on BMI z-score; Model 3 estimated the effects of p1, p2, p3, and p4 on BMI z-score, however, only legislation that specifically targeted youth were included; Model 4 estimated the effects of p1, p2, p3, and p4 on BMI z-score, however, only legislation that was implemented before 2015 was included.

*Note*. Estimates and corresponding standard errors and p-values of multilevel regression models with imputed data (m = 20): random intercepts.

The results of exploratory analyses showed that physical activity-related legislation enacted before 2015 had a statistically significant, but very modest association with the prevalence of overweight/obesity among adolescents in a state (Table 2). More specifically, a larger number of physical activity-related legislation was associated with lower overweight/obesity prevalence among adolescents (*b* = −0.002, *p* = .042). The *p*-value exceeded 0.05 after correcting for multiple comparisons.

**Table 2:** Results of linear regression models.

	Model 1			Model 2			Model 3			Model 4		
	Est	SE	p-value	Est	SE	p-value	Est	SE	p-value	Est	SE	p-value
Intercept	0.307	0.013	<0.001	0.307	0.013	<0.001	0.304	0.012	<0.001	0.307	0.013	<0.001
P0: Total	0.000	0.000	0.927									
P1: Nutrition				0.002	0.001	0.216	−0.002	0.002	0.343	0.001	0.001	0.314
P2: PA				−0.002	0.001	0.057	0.000	0.003	0.927	−0.002	0.001	0.042*
P3: Combined				0.003	0.002	0.170	0.003	0.003	0.381	0.004	0.002	0.097
P4: Other				−0.001	0.002	0.412	0.013	0.013	0.328	−0.001	0.001	0.554

*Note*. Model 1 estimated the effect of the total number of laws p0 on BMI z-score; Model 2 estimated the effects of p1, p2, p3, and p4 on BMI z-score; Model 3 estimated the effects of p1, p2, p3, and p4 on BMI z-score, however, only legislation that specifically targeted youth were included; Model 4 estimated the effects of p1, p2, p3, and p4 on BMI z-score, however, only legislation that was implemented before 2015 was included.

*Note*. Estimates and corresponding standard errors and p-values of exploratory linear regression models with proportion overweight/obesity as dependent variable.

## 4 Discussion

The aim of this study was to test whether differences in numbers of obesity-related legislation were associated with adolescents’ BMIz and prevalence of overweight/obesity. Main analyses showed that there were no statistically significant associations between state-level legislation and adolescent BMIz, however, exploratory analyses did show a statistically significant effect: a higher number of state-level physical activity-related legislation implemented before 2015 was statistically significantly associated with a lower proportion overweight/obesity among adolescents in that state, before correcting for multiple comparisons. In detail, implementing ten more laws would be associated with 2% fewer adolescents with overweight/obesity, but only after a few years, i.e., three years according to our analyses. It seems likely that it takes time before obesity-related legislation might affect overweight/obesity prevalence, as the period of time between the enactment of a law and its execution in the real world (e.g., improving a side walk, or changing the contents of vending machines at schools) might be considerable. Likewise, the lag between the execution of legislation and the noticeable effects it has on adolescents might be considerable; it takes a time before someone notices that the sidewalk has improved and that it may now be safe to cycle or walk to school.

How can we explain that an intervention or measure is associated with lower overweight/obesity prevalence but not with decreased BMI? Previous research has shown that the prevalence of overweight/obesity had changed, while average or median BMI remained practically the same [29], [30]. This was likely due to the group of people who were borderline overweight, i.e., those who do not severely impact the group average but contribute – potentially crucially - to the weight status categories, i.e., normal weight or overweight/obese. This goes to suggest that the association between the quantity of legislation and weight-related outcomes might differ for distinct groups of individuals, and might perhaps not be linear either; it seems unlikely that installing 100 laws would be associated with 20% fewer adolescents with overweight/obesity and installing 200 laws with 40% fewer adolescents with overweight/obesity, et cetera. To speculate, we would expect that the association might have the form of an inverse S-shaped curve, hypothesizing that a very small number of laws would not affect overweight/obesity prevalence, as these hardly constitute a change in the obesogenic environment. But a larger quantity of laws can, and we would expect that the more laws are installed, the more enforcing the changes in the obesogenic environment are, until the effects level off. Here, we only modeled linear effects to establish first insights into associations between quantity of laws and weight-related outcomes but future studies are needed where non-linear effects are examined to draw firmer conclusions about potential levelling-off. The results of this study might also explain why previous studies have not found any statistically significant associations [18], [22]; these studies considered BMI as dependent variable and did not take into account overweight/obesity prevalence.

Furthermore, only physical-activity related laws were associated with weight-related outcomes (and only prior to correction for multiple testing was applied) – not nutrition-related or combined legislation. This is somewhat surprising, as combined interventions tend to be more effective than single-focus interventions [32], [33]. As the nutrition-related category contained the most laws, it is unlikely that number of laws per category played a major role. Compared to other groups in society, adolescents might relatively easily increase their physical activity, as they often have mandatory exercise classes at school and are more frequently participating in sports teams, whereas changing dietary habits requires more intrinsic motivation. Moreover, the dietary habits of adolescents usually depend to a large part on the groceries their parents purchase.

Finally, we analyzed the effects of the quantity of obesity-related legislation on the average BMI and overweight/obesity prevalence of adolescents, because previous research showed that the most frequently installed obesity-related laws explicitly target youth [18]. Notably, in our data, only 436 out of 1475 (30%) enacted laws explicitly targeted youth, which could potentially explain why there was no statistically significant association with adolescent BMI, and merely one statistically significant association with overweight/obesity prevalence before correcting for multiple comparisons. Another possibility is that the effects of the installed legislation have a longer lag period, thus, it might be that future youth do experience benefits from a greater number of obesity-related laws, or that the youth included in this research will experience greater effects as they grow up to be young adults. Therefore, examining the association between installed legislation and the population of young adults specifically might be a valuable addition to the existing literature on the quantity of obesity-related legislation.

### 4.1 Limitations and implications for future research

Height and weight data used in our analyses were based on self-reports. Although YRBS questions generally have good test–retest reliability [34], weight is known to be subject to biased self-reporting [35], which might have influenced our results if specific subgroups are more likely to under- or over-report their weight than others. Furthermore, height and weight data were only available from participants in 33 states. Our results might have been different if the remaining states were included in the analyses, however, this would imply that adolescents in states with certain obesity-related characteristics are less likely to participate in the YRBS. Future research might replicate and extend this study’s findings by employing objectively measured anthropometric data and by including data of the 17 states that were absent in the YRBS 2017 data file.

With regards to the use of the YRBS several drawbacks of this data set could be mentioned that are outlined elsewhere [36]. For example, the YRBS is administered at schools, therefore excluding adolescents that do not (regularly) attend school. This is of relevance, because many health risk behaviors are more prevalent among youth that do not attend school. Moreover, we cannot be certain that the CDC’s data set on obesity-related legislation is complete.

Previous studies have shown that individual-level characteristics such as SES and ethnicity might interact with policy-level interventions [16], [37]. Given that the level of analysis in the present study was the state rather than the individual, and there was no reason to assume that state-level SES or ratio of ethnic groups would impact the association, we did not include SES or ethnicity here. That said, future research could explore how characteristics of states might affect how the quantity of obesity-related legislation leads to changes in the population. Furthermore, for this research, we assumed that all laws have equal impact on weight-related outcomes, however, it might be that some laws have a more severe impact on weight-related outcomes than others. For instance, earlier enacted laws might have more impact than later implemented laws, or laws that are supported by scientific research might have a greater effect on weight-related outcomes than laws that cannot be supported by scientific knowledge. Future studies could focus on the design of such a weighting system that informs which laws should be weighted more heavily than others.

Finally, studies on associations between policy-level interventions and individual-level health outcomes emphasize the difficulty of investigating this association [37], [38], [39], [40], [41]. Many different frameworks and methodologies [42], [43] have been proposed and tested, but consensus on how to evaluate policy-level interventions has not yet been reached, which highlights the relevance for future research of clearly defined and described concepts and methods, to facilitate comparison between studies, and eventually to evaluate the evidence base for the effectiveness of policy-level interventions to prevent and reduce (childhood) obesity.

## 5 Conclusions

The findings of this study contribute to the existing literature on the quantity of policy-level interventions and its effect on weight outcomes. Over the years, the number of introduced and installed legislation has grown [18], [19], [20], suggesting that the attention paid and effort undertaken to combat (childhood) obesity at policy-level has increased. The current study suggests a possible association between the quantity of obesity-related legislation and adolescent weight-outcomes. The results emphasize the importance of policy-level interventions as a strategy for combatting youth obesity, and show the value of including both BMI and prevalence of overweight/obesity.

### CRediT authorship contribution statement

**Annita Kobes:** Conceptualization, Methodology, Formal analysis, Writing – original draft, Visualization. **Tina Kretschmer:** Conceptualization, Methodology, Writing – review & editing, Supervision. **Margaretha C. Timmerman:** Conceptualization, Writing – review & editing, Supervision.

## Declaration of Competing Interest

The authors declare that they have no known competing financial interests or personal relationships that could have appeared to influence the work reported in this paper.
