# Supplementary material for: The association between obesity-related legislation in the United States and adolescents’ weight
Source: Health Policy Open. 2021 Dec 1;3:100056. doi: 10.1016/j.hpopen.2021.100056 (PMC10297798; doi:10.1016/j.hpopen.2021.100056)
Supplement: Supplementary data 1 [file mmc1.docx]

**Table S1**

*Results of random intercepts + slopes multilevel regression models with imputed data*

|  | Model 1 | | | Model 2 | | | Model 3 | | | Model 4 | | |
| --- | --- | --- | --- | --- | --- | --- | --- | --- | --- | --- | --- | --- |
|  | Est | SE | *p*-value | Est | SE | *p*-value | Est | SE | *p*-value | Est | SE | *p*-value |
| Intercept | 0.509 | 0.077 | <.001 | 0.564 | 0.031 | .004 | 0.557 | 0.02 | <.001 | 0.564 | 0.031 | .004 |
| *P0:* Total | 0.001 | 0.001 | .748 |  |  |  |  |  |  |  |  |  |
| *P1:* Nutrition |  |  |  | 0.001 | 0.004 | .745 | -0.001 | 0.005 | .874 | 0.001 | 0.004 | .745 |
| *P2:* PA |  |  |  | -0.003 | 0.003 | .443 | 0.006 | 0.006 | .373 | -0.003 | 0.003 | .443 |
| *P3:* Combined |  |  |  | 0.006 | 0.006 | .561 | 0.003 | 0.006 | .643 | 0.006 | 0.006 | .561 |
| *P4:* Other |  |  |  | -0.001 | 0.004 | .900 | 0.008 | 0.041 | .854 | -0.001 | 0.004 | .900 |

*Note.* Model 1 estimated the effect of the total number of laws *p0* on BMI z-score; Model 2 estimated the effects of *p1*, *p2*, *p3*, and *p4* on BMI z-score; Model 3 estimated the effects of *p1*, *p2*, *p3*, and *p4* on BMI z-score, however, only legislation that specifically targeted youth were included; Model 4 estimated the effects of *p1*, *p2*, *p3*, and *p4* on BMI z-score, however, only legislation that was implemented before 2015 was included.

*Note*. Estimates and corresponding standard errors and p-values of multilevel regression models: random intercepts and random slopes

**Table S2**

*Results of random intercepts models with non-imputed data*

|  | Model 1 | | | Model 2 | | | Model 3 | | | Model 4 | | |
| --- | --- | --- | --- | --- | --- | --- | --- | --- | --- | --- | --- | --- |
|  | Est | SE | *p*-value | Est | SE | *p*-value | Est | SE | *p*-value | Est | SE | *p*-value |
| Intercept | 0.547 | 0.032 | <.001 | 0.547 | 0.033 | <.001 | 0.544 | 0.029 | <.001 | 0.547 | 0.033 | <.001 |
| *P0:* Total | 0.000 | 0.001 | .748 |  |  |  |  |  |  |  |  |  |
| *P1:* Nutrition |  |  |  | 0.004 | 0.003 | .171 | -0.006 | 0.029 | .263 | 0.004 | 0.003 | .171 |
| *P2:* PA |  |  |  | -0.005 | 0.002 | .078 | 0.004 | 0.005 | .608 | -0.004 | 0.003 | .078 |
| *P3:* Combined |  |  |  | 0.007 | 0.006 | .286 | 0.006 | 0.007 | .437 | 0.007 | 0.006 | .286 |
| *P4:* Other |  |  |  | -0.003 | 0.005 | .498 | 0.014 | 0.033 | .670 | -0.003 | 0.005 | .498 |

*Note.* Model 1 estimated the effect of the total number of laws *p0* on BMI z-score; Model 2 estimated the effects of *p1*, *p2*, *p3*, and *p4* on BMI z-score; Model 3 estimated the effects of *p1*, *p2*, *p3*, and *p4* on BMI z-score, however, only legislation that specifically targeted youth were included; Model 4 estimated the effects of *p1*, *p2*, *p3*, and *p4* on BMI z-score, however, only legislation that was implemented before 2015 was included.

*Note.* Estimates and corresponding standard errors and p-values of multilevel regression models on not-imputed data: random intercepts
